# Novel Vitronectin Variations and Their Comparative Analysis in Six Porcine Breeds

**DOI:** 10.3390/ani9080520

**Published:** 2019-08-02

**Authors:** Wei Yan, Xutin Zhao, Juyin Li, Long Cheng, Yanqing Li

**Affiliations:** 1 School of Animal Science and Technology, Jiangsu Agri-animal Husbandry Vocational College, Taizhou 225300, China; 2 Faculty of Veterinary and Agricultural Sciences, The University of Melbourne, Dookie Campus, VIC 3647, Australia; 3 IPIG Management & Consulting Company Limited, Guangzhou 511457, China

**Keywords:** Vitronectin, feed efficiency, pigs

## Abstract

**Simple Summary:**

Feed efficiency strongly influences the productivity and profitability in modern swine farming enterprises. Residual feed intake (RFI) as a measure of feed efficiency has been extensively studied, which is defined as the differing value between observed feed intake and expected feed intake based on growth and backfat. There is a potential that the porcine vitronectin plays a role in RFI through affecting the energy metabolism within muscles. Using sequencing technology, we found that rich variations and amino acid changes existed in key regions of vitronectin. In addition, a difference in variant frequencies was observed between local Chinese breeds (Sujiang, Jiangqu-hai, and Jiaxing-black) and three well characterized breeds (American Duroc, Canadian Duroc, and Berkshire) derived from the overseas. We suggest that the porcine vitronectin gene can be studied further and may be developed as a gene marker to select high feed-efficiency swines.

**Abstract:**

Vitronectin plays a role in the blood homeostasis and has been implicated in cell adhesion, migration, and proliferation. Vitronectin has a potential role affecting the residual feed intake (RFI) or feeding efficiency in swine production. Its variations have not been reported in Chinese swine breeds. In this study, two regions of porcine vitronectin were analyzed using PCR and sequencing. The sequence analysis revealed thirteen nucleotide substitutions in region 1 (exon 2- exon 3) and three nucleotide substitutions in region 2 (exon 5- intron 5), which would result in five amino acid changes (p.Ala52Thr, p.Leu94Pro, p.Leu94Gln, p.Gln94Pro, and p.Glu126Gly). In region 1, c.156C/T, c.281A/T, and c.377A/G were the most common (at a total frequency of 49.3%, 31.3% and 31.9% respectively), whereas c.153C/T and c.180C/G were rare (at a total frequency of 1.39%). In region 2, c.597 + 12A/G was the most common (at a total frequency of 39.6%), followed by c.597 + 15A/G (at a total frequency of 31.3%) and c.459A/G (at a total frequency of 16.0%). There was a difference (*p* < 0.05) in variant frequencies between Chinese breeds and overseas breeds. These results indicate that the porcine vitronectin gene is polymorphic and suggest further analysis is required to see if the variation detected affects RFI or feed efficiency in swines.

## 1. Introduction

Vitronectin, abbreviated as VTN, is also known as s-protein or serum diffusion factor. It is a multifunctional glycoprotein [1,2]. VTN in mammals is widely distributed in the serum, the plasma and the blood platelet alpha particle [3], which is mainly synthesized and secreted in liver tissue, and it was also observed in the urethra and retina tissues [4,5]. It functions as a facilitator of cell adhesion, migration, and proliferation [6].

The vitronectin gene has been identified in various biological species, including humans, mice, pigs, sheep, cattle, chickens, and rabbits, and across these species the porcine vitronectin gene has about 80% homology with humans, mice, and rabbits [7]. The porcine vitronectin gene is located in the genome region (g.44650622–g.44653871) of chromosome 12, including eight exons interrupted by seven introns. Two spliced variants with sizes of 1377 bp and 1161 bp were found which encoded 458 amino acids and 386 amino acids, respectively. The single nucleotide substitutions have been reported in several above species.

In humans and mice, variations in the vitronectin gene have been associated with thrombogenesis [8], atherosclerosis [9] and the coronary heart disease [2]. To date, there have been limited reports about variations in the vitronectin gene affecting the production traits in domestic animals, particularly in swines. However, a previous study attracted our interest in the porcine vitronectin gene, as it was involved in the residual feed intake (RFI) as a measure of feed efficiency, which is defined as the differing value between observed feed intake and expected feed intake based on growth and backfat [10]. The observation of higher vitronectin protein expression in the serum of pigs with low RFI reflects a potential that the porcine vitronectin plays a role in RFI through affecting the energy metabolism within muscles [11]. Therefore, studies on variations of porcine vitronectin gene at the DNA level contribute to understanding its potential role in altering RFI. In other domestic animals, genes related to RFI in cattle were also found, including the methylcrotonoyl-CoA carboxylase 1 (MCCC1), the aldehyde oxidase 1 (AOX1) and the propionyl-CoA carboxylase alpha subunit (PCCA)) [12].

In this study, we used PCR and sequencing technology to analyze the genetic variation in two key regions (exon 2-exon 3 and exon 5-intron 5) of vitronectin, which include key amino acids of functional binding regions, in three local breeds in China (Sujiang, Jiangqu-hai, and Jiaxing-black) and three overseas breeds (American Duroc, Canadian Duroc, and Berkshire).

## 2. Materials and Methods

### 2.1. Animals and Sampling

All experimental procedures were performed in accordance with the animal welfare Jan 1988 (Chinese Government). A total of 144 ear tissue samples were collected from individual animal. Several small pieces of ear tissue were cut out using the disinfection scissor and kept in the sampling tube prior to analysis. The scissor was cleaned every time using 75% alcohol before sampling again. Individual animals from each breed (Table 1) belong to purebred boars of four unrelated sire lines and reared on the same farm. They were part of a performance test of boars twice a year supported by porcine quality supervision and testing center of Ministry of Agriculture in China. The number and source of samples are shown in Table 1.

### 2.2. DNA Extraction and PCR Amplification

The DNA extraction kit (Tiangen, China) was used for extracting genomic DNA.

Two variable regions of vitronectin were analyzed using PCR amplification and sequencing technology. Information on these amplified regions is shown in Table 2.

Four sets of PCR primers were designed for amplification of variable regions using DNAMAN (version 5.2.10, Lynnon BioSoft, Vaudreuil, Que, Canada) based on the published porcine vitronectin DNA sequence (Ensembl:ENSSSCG00000027801) and cDNA sequence (Ensembl:ENSSSCT00000025790.2). These primers are shown in Table 2 and were synthesized by General Biosystems company (Anhui, China).

Amplifications were performed in a 25μL reaction containing deletion 1 μL of DNA, 1 μL of each primer (10 μm), 12.5 μL of 2 × reaction mix (Tiangen, Shanghai, China), 0.25 μL of Taq DNA polymerase (0.5U) (Tiangen, Shanghai, China) and 9.25 μL of ddH_2_O.

The thermal profiles for the two regions amplified consisted of 3 min at 94 °C, followed by 30 cycles of 30 s at 94 °C, 30 s at 58 °C and 1 min at 72 °C, with a final extension of 5 mins at 72 °C. Amplification was carried out in 9700 thermal cyclers (ABI, USA). Amplifications were visualized by electrophoresis in 1% agarose gels (Tiangen, Shanghai, China), using 1 × TBE buffer (89 mm Tris, 89 mm boric acid, 2 mm Na_2_EDTA) containing 200 ng/mL of ethidium bromide.

### 2.3. Sequencing of Amplicons and Sequence Analysis

Amplicons were directly sequenced at the General Biosystems company (Hefei, China), deletion and every sample was sequenced from two directions to ensure the results are reliable.

Sequence alignment, translations, and comparisons were carried out using DNAMAN (version 5.2.10, Lynnon BioSoft, Vaudreuil, Que, Canada).

### 2.4. Statistical Analysis

Variable frequencies (>10% at a total frequency) in different breeds were analyzed using a chi-square test in SPSS version 24.0 (SPSS Science Inc., Chicago, IL, USA) with a significance level declared at *p* < 0.05.

## 3. Results

### 3.1. Identification of the Variation and Amino Acid Change in the Amplified Regions

**Figure 1:**
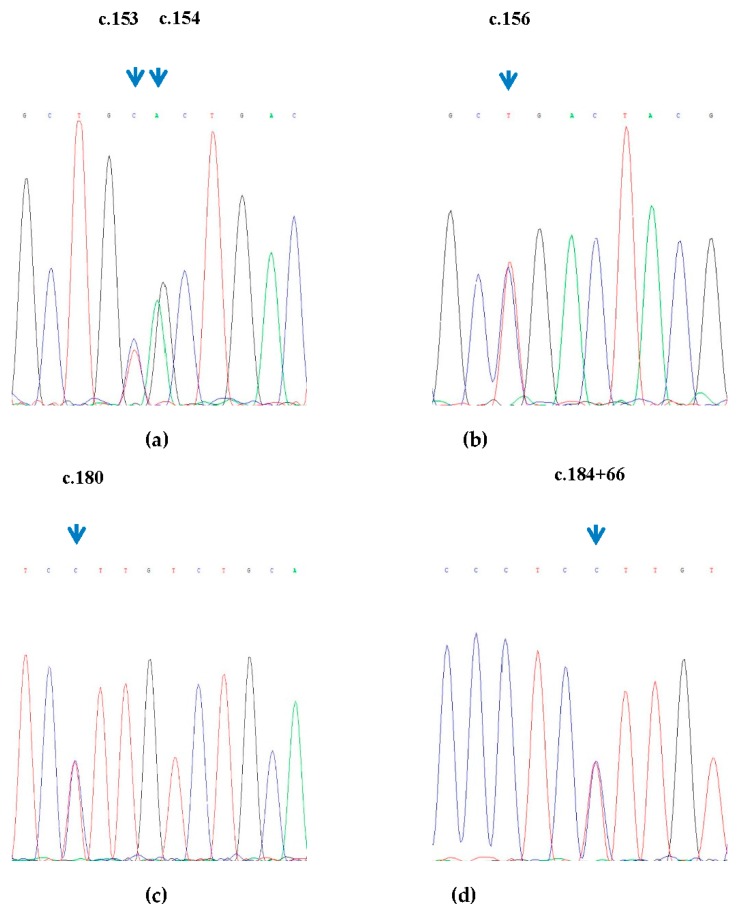

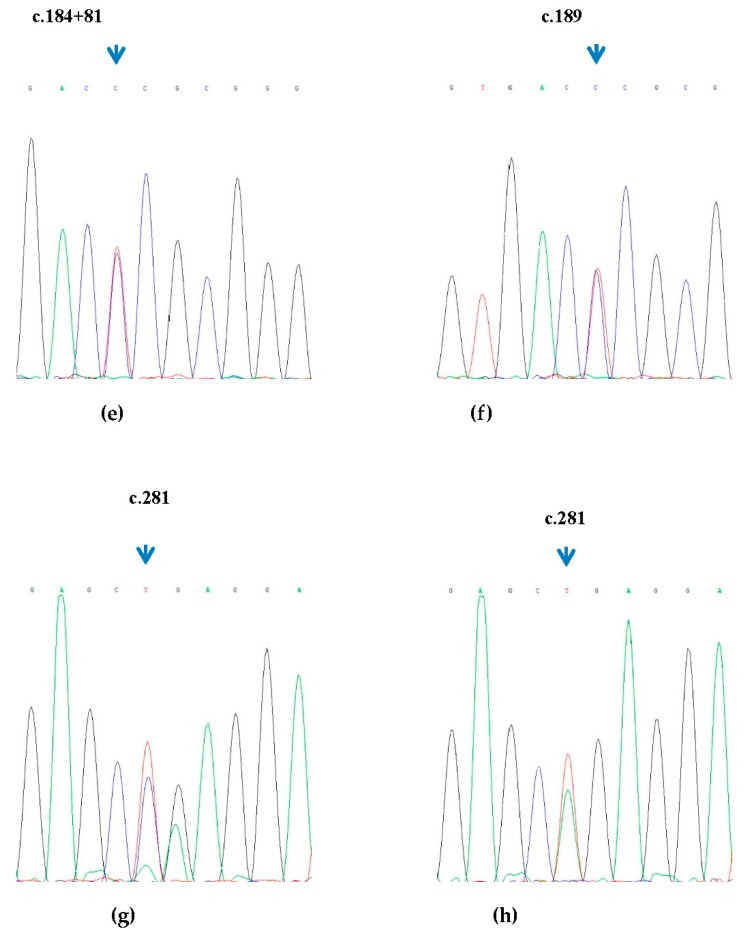

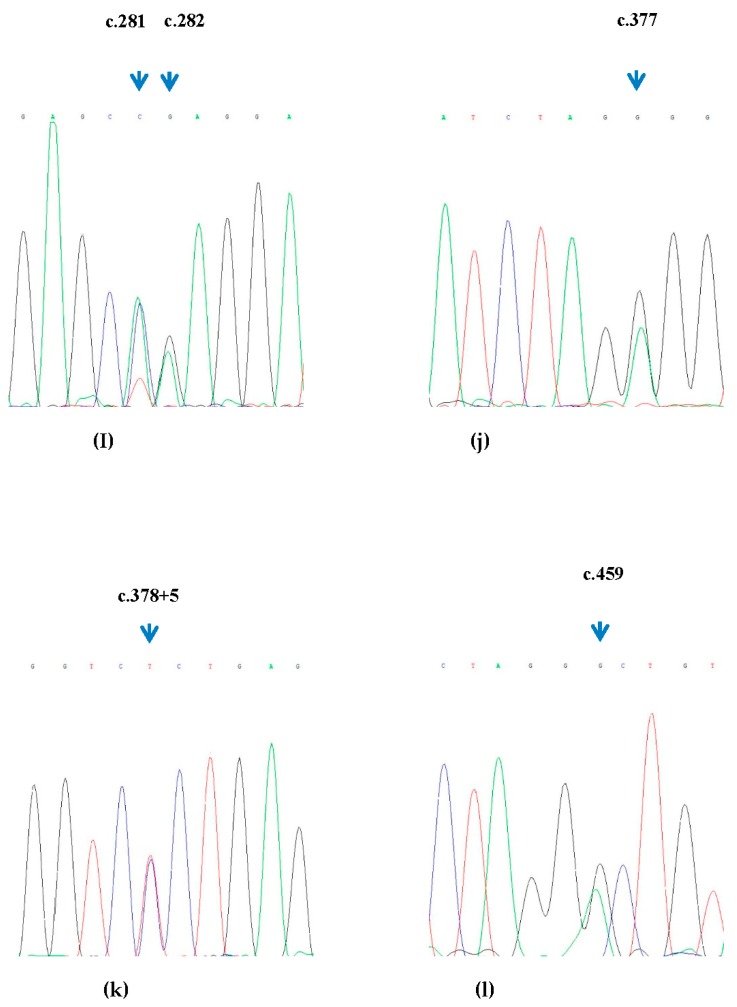

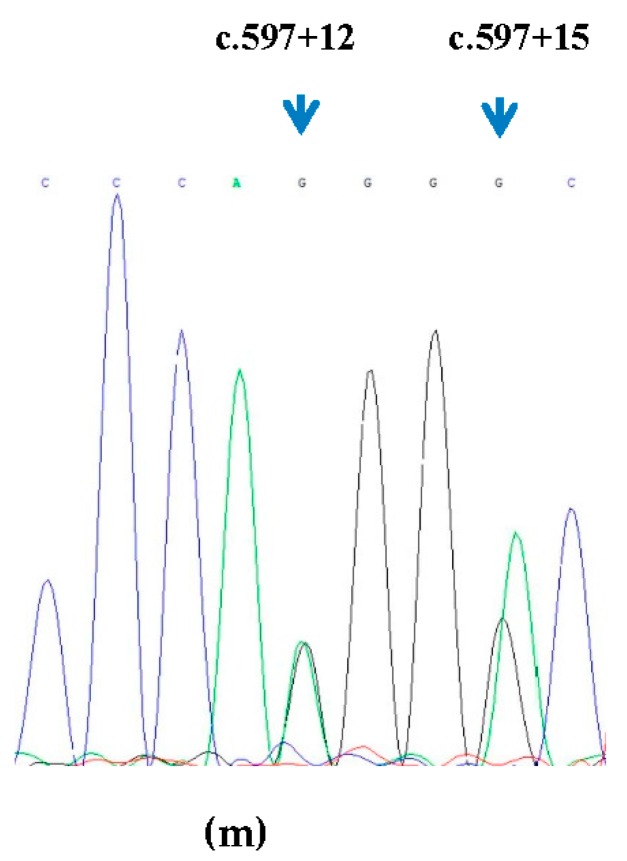
Sequencing maps of SNPs. (**a**) The sequencing map of SNPs (c.153C/T and c.154G/A). (**b**) The sequencing map of SNP (c.156C/T). (**c**) The sequencing map of SNP (c.180C/G). (**d**) The sequencing map of SNP (c.184 + 66C/T). (**e**) The sequencing map of SNP (c.184 + 81C/T). (**f**) The sequencing map of SNP (c.189C/T). (**g**) The sequencing map of SNP (c.281T/C). (**h**) The sequencing map of SNP (c.281T/A). (**i**) The sequencing map of SNPs (c.281C/A and c.282A/G). (**j**) The sequencing map of SNP(c.377A/G). (**k**) The sequencing map of SNP (c.378 + 5C/T). (**l**) The sequencing map of SNP (c.459A/G). (**m**) The sequencing map of SNPs (c.597 + 12A/G and c.597 + 15A/G).

In the amplified regions, a total of sixteen nucleotide substitutions were identified upon sequencing (Table 3 and Figure 1). It is notable that thirteen nucleotide substitutions are novel and several substitutions in these positions (c.154G/A, c.281T/C, c.281A/T, c.281A/C, and c.377A/G) would result in amino acid changes (Table 3).

### 3.2. Frequencies of Variations in Different Breeds

Frequencies of all nucleotide substitutions were analyzed in six breeds (Table 4). Single nucleotide substitutions (c.154G/A) were only observed in Jiangqu-hai, Canadian Duroc and Berkshire breeds with a total frequency of 10.4%. Three types of single nucleotide substitutions were found in the position (c.281) and the substitution (c.281A/T) was found to show the highest frequency of 31.3%, which were not observed in Canadian Duroc and Berkshire breeds. The c.281A/T substitution was only observed in Chinese breeds (Jiaxing-black, Jiangqu-hai, and Sujiang) with a total frequency of 31.9%, but not found in overseas breeds. The substitution (c.156C/T) was observed in five breeds (Jiaxing-hei, Jiangqu-hai, Sujiang, American Duroc, and Canadian Duroc) with the highest total frequency of 49.3%, but not found in Berkshire breed.

### 3.3. A difference of Variable Frequencies in Various Breeds

There were frequency differences (*p* < 0.05) in five positions (c.156C/T, c.281T/C, c.281A/T, c.282A/G and c.459A/G) among six breeds (Table 5). In the position (c.156C/T), the differences (*p* < 0.05) were all observed in Chinese breeds and overseas breeds. Jiaxing-black, Jiangqu-hai, and Canadian Duroc were found to have high frequencies of 79.2%, 79.2%, and 62.5%, respectively. In the position (c.281), the T/C substitution with a frequency of 45.8% was the most common in Sujiang breed (*p* < 0.05), but the A/T substitution with a frequency of 79.2% was common in Jiaxing-black and Jiangqu-hai breeds (*p* < 0.05). The c.282A/G substitution was the most common in Canadian Duroc with a frequency of 33.3% comparing with other breeds (*p* < 0.05). In the position (c.459A/G), the A/G substitution was the most common in Berkshire breed with the frequency of 62.5% (*p* < 0.05), followed by Jianghu-hai and Canadian Duroc breed with a frequency of 16.7%.

## 4. Discussion

Thirteen nucleotide substitutions were firstly found in exon 2, exon 3, and exon 5, as well as the introns (i.e., intron 2, intron 3 and intron 5) of porcine vitronectin in this study. The variant frequencies were observed in different breeds derived from China and overseas.

Eleven nucleotide substitutions (Table 3) were located in the exon 2, exon 3 and exon 5 and five nucleotide substitutions putatively result in amino acid changes (Table 3). It is notable that these are missense mutations, which may affect protein function or stability and cause variation in phenotype. The polarity changes of amino acids were observed in three positions, including the p.Ala52Thr, p.Leu94Gln and p.Gln94Pro, but the change of charge property was observed only in the position p.Glu126Gly. Several key functional regions of vitronectin have been identified, including the thrombin-antithrombin complex 3 binding region extending from the 53th to the 64th amino acid, the hemopexin binding region extending from the 132th to 459th amino acid [13] and the RGD region involving the 45th, 46^th^, and 47th amino acid, which combined the integrin to facilitate the cell adhesion [14]. It has been reported that the change of 50th and 57th amino acid next to the RGD region affected the RGD function strongly [15]. Therefore, there is a potential that the non-synonymous mutation (c.154G/A) resulting in amino acid change (p.Ala52Thr) which is adjacent to the key regions, would affect the vitronectin function. The 52th amino acid site (p.Ala52Thr) may be O-glycosylated giving rise to the change of dimensional folding on the tertiary or quaternary structure of protein, as well as of the protein stability. Porcine vitronectin gene, therefore, might be quite polymorphic, which is interesting for further discussing how this variation came about or is sustained in swines.

The variant frequencies for different breeds were calculated in this study. Some variations were not observed in some of the breeds, but caution is needed in interpreting this result, as the swine types were not chosen so as to necessarily be representative of the breed as a whole. Given this constraint, the difference between frequencies in Chinese breeds and overseas breeds was, however, large because these Chinese breeds are native with limited artificial selection during breeding program, and they have rich genetic diversity [16,17]. In contrast, the artificial selection was carried out on the overseas breeds investigated in this study for a long time and the sustaining selection pressure may have reduced some variant frequencies. Sujiang breed was originally developed as a Jiangqu hai-American Duroc cross, so these breeds owned a common background genetically, but the frequencies for these breeds appear to be quite different. The difference between American Duroc and Berkshire seemed not to be small. American Duroc is mainly bred for the purpose of high growth speed and reduced backfat thickness [18], while Berkshire is mainly bred for the meat quality trait, such as the content of IMF [19]. Therefore, the sustaining artificial selection pressure based on different breeding targets and feeding environment may affect the vitronectin gene region.

In this study, the genetic difference at vitronectin between American Duroc and each of other breeds was pronounced, as the variant frequencies for American Duroc were found to be null or lower than other breeds, and the highest frequency (29.1%) was only found in the position c.156C/T. This suggests that the variation in vitronectin might underpin some of the differences between American Duroc and each of other breeds. However, more research is needed on out-bred porcine populations to support this finding, as firstly, the random drift may affect the observed frequency, and secondly, these observed frequencies in vitronectin may simply reflect a founder effect or some other bottleneck in the selection of these American Duroc populations. Nevertheless, the current study result indicates more work is needed to understand the vitronectin impact on the production traits in swine.

## 5. Conclusions

In conclusion, thirteen novel variations in exon 2, exon 3 and exon 5/ intron 2, intron3 and intron 5 were identified, which would result in amino acid change (p.Ala52Thr, p.Leu94Pro, p.Leu94Gln, p.Gln94Pro, and p.Glu126Gly), might affect the function of vitronectin in swines. Furthermore, the differences of variant frequencies were found to be variant in 144 pigs derived from six breeds. Therefore, the porcine vitronectin gene is polymorphic and appears to have different genetic characteristics at vitronectin among six breeds.

## Figures and Tables

**Table 1:** Number of individual animals from six breeds used in this study.

Breed Abbreviation	Breed Name	Sampling Location	Number (n)
MX	American Duroc	Sampled in Hunan, China	24
JX	Canadian Duroc	Sampled in Jiangsu, China	24
BKX	Berkshire	Sampled in Jiangsu, China	24
JQH	Jiangqu-hai	Sampled in Jiangsu, China	24
SJ	Sujiang	Sampled in Jiangsu, China	24
JXH	Jiaxing-black	Sampled in Zhejiang, China	24
Total			144

**Table 2:** The primer sequences for amplicons.

	Primer Sequence (5′–3′)	Amplicon Size	Amplified Region
Region 1	Up: GCTGTCATACTCCCTCTCCA	498 bp	Spanning a region of exon 2 and exon 3
	Dn: TCTGCCATTCCAGTCACCT		
Region 2	Up: ACTGGAATGGCAGACCTTG	253 bp	Spanning a region of exon 5 and intron 5
	Dn: AGATAGCCTTGACCCTGACC		

**Table 3:** Variation and amino acid change in amplified regions.

Gene	Variable Region	Position ^a^	Amino Acid Change
Vitronectin	Exon 2	c.153C/T *	No change
		c.154G/A *	Ala52Thr
		c.156C/T *	No change
		c.180C/G *	No change
	Intron 2	c.184 + 66C/T *	/
		c.184 + 81C/T *	/
	Exon 3	c.189C/T *	No change
		c.281T/C	Leu94Pro
		c.281A/T	Leu94Gln
		c.281A/C	Gln94Pro
		c.282A/G *	No change
		c.377A/G *	Glu126Gly
	Intron 3	c.378 + 5C/T *	/
	Exon 5	c.459A/G *	No change
	Intron 5	c.597 + 12A/G *	/
		c.597 + 15A/G *	/

* Novel single nucleotide substitution. ^a^ The numbering of positions follows the guidelines presented on https://www.hgvs.org/content/guidelines.

**Table 4:** Frequencies of the porcine variations in six breeds.

	Breed	JiaXing-Black (Frequency) (*n* = 24)	Jiangqu-Hai (Frequency) (*n* = 24)	SuJiang (Frequency) (*n* = 24)	American Duroc (Frequency) (*n* = 24)	Canadian Duroc (Frequency) (*n* = 24)	Berkshire (Frequency) (*n* = 24)	Total (Frequency) (*n* = 144)
Position								
c.153C/T		0.00%	0.00%	0.00%	0.00%	0.00%	8.3% (2)	1.39%
c.154G/A		0.00%	29.1% (7)	0.00%	0.00%	25.0% (6)	8.3% (2)	10.4%
c.156C/T		79.2% (19)	79.2% (19)	45.8% (11)	29.1% (7)	62.5% (15)	0.00%	49.3%
c.180C/G		0.00%	0.00%	0.00%	0.00%	0.00%	8.3% (2)	1.39%
c.184 + 66C/T		0.00%	29.1% (7)	0.00%	0.00%	29.1% (7)	8.3% (2)	11.1%
c.184 + 81C/T		79.2% (19)	29.1% (7)	0.00%	16.7% (4)	0.00%	0.00%	20.8%
c.189C/T		0.00%	33.3% (8)	45.8% (11)	0.00%	0.00%	0.00%	13.2%
c.281T/C		0.00%	0.00%	45.8% (11)	0.00%	33.3% (8)	8.3% (2)	14.6%
c.281A/T		79.2% (19)	79.2% (19)	12.5% (3)	16.7% (4)	0.00%	0.00%	31.3%
c.281A/C		0.00%	0.00%	16.7% (4)	8.3% (2)	0.00%	0.00%	4.17%
c.282A/G		0.00%	33.3% (8)	45.8% (11)	8.3% (2)	33.3% (8)	8.3% (2)	21.5%
c.377A/G		79.2% (19)	62.5% (15)	50.0% (12)	0.00%	0.00%	0.00%	31.9%
c.378 + 5C/T		0.00%	50.0% (12)	50.0% (12)	8.3% (2)	62.5% (15)	0.00%	28.5%
c.459A/G		0.00%	16.7% (4)	0.00%	0.00%	16.7% (4)	62.5% (15)	16.0%
c.597 + 12A/G		79.2% (19)	12.5% (3)	45.8% (11)	8.3% (2)	91.7% (22)	0.00%	39.6%
c.597 + 15A/G		66.7% (16)	29.1% (7)	33.3% (8)	0.00%	50.0% (12)	8.3% (2)	31.3%

**Table 5:** A difference of variable frequencies in various breeds.

Sites	Breeds (Frequency %)	*p* Value	Breeds (Frequency %)	*p* Value	Breeds (Frequency %)	*p* Value
c.154G/A	Jiangqu-Hai (29.1%)	*p* > 0.05	Canadian Duroc (25.0%)	*p* > 0.05	/	/
	Canadian Duroc (25.0%)		Berkshire (8.3%)		/	/
	Berkshire (8.3%)		/	/	/	/
c.156C/T	Jiaxing-black (79.2%)	*p* < 0.05	JiaXing-Black (79.2%)	*p* < 0.05	American Duroc (29.1%)	*p* < 0.05
	Jiangqu-Hai (79.2%)		Jiangqu-Hai (79.2%)		Canadian Duroc (62.5%)	
	Sujiang (45.8%)		SuJiang (45.8%)		/	/
	American Duroc (29.1%)		/	/	/	/
	Canadian Duroc (62.5%)		/	/	/	/
c.281T/C	Sujiang (45.8%)	*p* < 0.05	Canadian Duroc (33.3%)	*p* < 0.05	/	/
	Canadian Duroc (33.3%)		Berkshire (8.3%)		/	/
	Berkshire (8.3%)		/	/	/	/
c.281A/T	Jiaxing-black (79.2%)	*p* < 0.05	JiaXing-Black (79.2%)	*p* < 0.05	/	/
	Jiangqu-Hai (79.2%)		Jiangqu-Hai (79.2%)		/	/
	Sujiang (12.5%)		SuJiang (12.5%)		/	/
	American Duroc (16.7%)		/	/	/	/
c.281A/C	Sujiang (16.7%)	*p* > 0.05	/	/	/	/
	American Duroc (8.3%)		/	/	/	/
c.282A/G	Jiangqu-Hai (33.3%)	*p* < 0.05	Jiangqu-Hai (33.3%)	*p* > 0.05	American Duroc (8.3%)	*p* < 0.05
	Sujiang (45.8%)		SuJiang (45.8%)		Canadian Duroc (33.3%)	
	American Duroc (8.3%)		/	/	Berkshire (8.3%)	
	Canadian Duroc (33.3%)		/	/	/	/
	Berkshire (8.3%)		/	/	/	/
c.377A/G	JiaXing-Black (79.2%)	*p* > 0.05	/	/	/	/
	Jiangqu-Hai (62.5%)		/	/	/	/
	SuJiang (50%)		/	/	/	/
c.459A/G	Jiangqu-Hai (16.7%)	*p* < 0.05	Canadian Duroc (16.7%)	*p* < 0.05	/	/
	Canadian Duroc (16.7%)		Berkshire (62.5%)		/	/
	Berkshire (62.5%)		/	/	/	/
